# Safety of Ustekinumab in Inflammatory Bowel Disease: Pooled Safety Analysis Through 5 Years in Crohn’s Disease and 4 Years in Ulcerative Colitis

**DOI:** 10.1093/ecco-jcc/jjae013

**Published:** 2024-02-04

**Authors:** Subrata Ghosh, Brian G Feagan, Elyssa Ott, Christopher Gasink, Bridget Godwin, Colleen Marano, Ye Miao, Tony Ma, Edward V Loftus, William J Sandborn, Silvio Danese, Maria T Abreu, Bruce E Sands

**Affiliations:** APC Microbiome Ireland, College of Medicine and Health, University College Cork, Cork, Ireland; Western University and Alimentiv Inc., London, ON, Canada; Formerly of Janssen Scientific Affairs, LLC, Horsham, PA, USA; Current affiliation: Merck & Co., Inc., Rahway, NJ, USA; Formerly of Janssen Scientific Affairs, LLC, Horsham, PA, USA; Current affiliation: Intercept Pharmaceuticals, Morristown, NJ, USA; Formerly of Janssen Scientific Affairs, LLC, Horsham, PA, USA; Janssen Research & Development, LLC, Spring House, PA, USA; Janssen Research & Development, LLC, Spring House, PA, USA; Janssen Research & Development, LLC, Spring House, PA, USA; Janssen Scientific Affairs, LLC, Horsham, PA, USA; Division of Gastroenterology and Hepatology, Mayo Clinic College of Medicine and Science, Rochester, MN, USA; Division of Gastroenterology, University of California San Diego, La Jolla, CA, USA; Mirador Therapeutics, San Diego, CA, USA; IRCCS Ospedale San Raffaele and University Vita-Salute San Raffaele Milano, Milan, Italy; University of Miami Miller School of Medicine, Miami, FL, USA; Dr. Henry D. Janowitz Division of Gastroenterology, Icahn School of Medicine at Mount Sinai, New York, NY, USA

**Keywords:** Inflammatory bowel disease, safety, ustekinumab

## Abstract

**Background and Aims:**

Previously published long-term safety data reported a favourable ustekinumab safety profile for the treatment of inflammatory bowel disease [IBD]. We present the final cumulative safety data from pooled ustekinumab IBD phase 2/3 clinical studies through 5 years in Crohn’s disease [CD] and 4 years in ulcerative colitis [UC].

**Methods:**

In phase 3 studies, patients received a single intravenous placebo or ustekinumab [130 mg or ~6 mg/kg] induction dose followed by subcutaneous maintenance doses of placebo or ustekinumab [90 mg q8w or q12w]. Analyses included all patients who received one dose of study treatment and included patients who were biologic-naïve and patients with a history of biologic failure. Safety outcomes are summarized and presented using number of events per 100 patient-years of follow-up and corresponding 95% confidence intervals.

**Results:**

In this final pooled safety analysis, 2575 patients were treated with ustekinumab with 4826 patient-years of follow-up. Rates of key safety events, including major adverse cardiac events and malignancies, were similar between placebo and ustekinumab or not higher for ustekinumab. Opportunistic infections, including tuberculosis, and malignancies were reported infrequently. Rates of key safety events in the IBD group were no higher in the ustekinumab group than in the placebo group for both patients who were biologic-naïve or who had a history of biologic failure. No lymphomas or cases of posterior reversible encephalopathy syndrome [formerly known as reversible posterior leukoencephalopathy syndrome] were reported.

**Conclusion:**

The final cumulative ustekinumab safety data through 5 years in CD and 4 years in UC demonstrated favourable safety compared to placebo and continue to support the well-established safety profile across all approved indications.

**Clinical trials.gov numbers:**

NCT00265122, NCT00771667, NCT01369329, NCT01369342, NCT01369355, NCT02407236

## Graphical Abstract

**Graphical Abstract:**
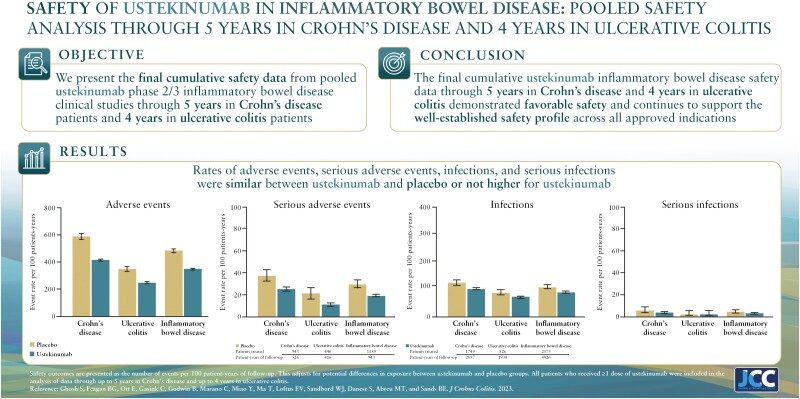

## 1. Introduction

Inflammatory bowel disease [IBD] is a chronic disease that requires long-term treatment and management. As therapy options for IBD increase, it is important to collect long-term safety data to inform therapeutic decisions for providers and patients. Integration and analysis of the ustekinumab safety data for Crohn’s disease [CD] and ulcerative colitis [UC] for a combined IBD population provides more complete information for treating-practitioners, facilitating benefit/risk assessment for their IBD treatment choices. This analysis increases the ability to detect safety signals, specifically for less frequent events (e.g. serious adverse events [SAEs] or serious infections). Because ustekinumab IBD indications uniquely employ intravenous [IV] induction, followed by subcutaneous [SC] maintenance dosing (90 mg every 8 [q8w] or 12 weeks [q12w]), these pooled safety analyses of all IBD phase 2/3 studies also provide important data examining whether these higher doses might have a different safety profile than the lower doses indicated for psoriatic diseases.^1^

A pooled analysis of safety data from six IBD studies with similar study designs [T07,^2^ CERTIFI,^3^ UNITI-1,^4^ UNITI-2,^4^ IM-UNITI,^4^ and UNIFI^5^] showed a favourable safety profile for ustekinumab through 1 year in UC and CD that was generally similar to placebo in patients with moderate to severe CD or UC.^6^ The IBD safety profile also aligned with previously published safety data across CD and psoriatic indications.^7^ Here we present the final compilation of cumulative safety data from ustekinumab phase 2/3 IBD clinical studies through 5 years in patients with CD and 4 years in patients with UC.

## 2. Methods

### 2.1. Trial designs

Data were pooled from six phase 2/3 IBD studies (five CD studies [T07,^2^ CERTIFI,^3^ two induction studies, UNITI-1 and UNITI-2, and one maintenance study, IM-UNITI^4^], and one phase 3 UC protocol consisting of separate induction and maintenance studies [UNIFI^5^]). All studies were registered at Clinicaltrials.gov: NCT00265122, NCT00771667, NCT01369329, NCT01369342, NCT01369355, and NCT02407236. Study details and trial designs have been previously published.^6^

Data presented in this analysis focus on long-term use with SC doses of placebo or ustekinumab 90 mg q8w or q12w. Overall baseline characteristics and safety results for the induction period and through 1 year have been previously published.^6^ Although, by study design, concomitant immunomodulators and corticosteroids were permitted for all IBD studies, patients were encouraged to taper corticosteroids starting at the beginning of maintenance treatment [i.e. 8 weeks after induction], and as a result, small numbers of patients were still receiving steroids after 1 year. Therefore, long-term safety analyses by concomitant steroids are not feasible. In phase 3, after the studies were unblinded to the investigative sites [occurred after week 44 analyses were completed for the last randomized patient], patients still receiving placebo were terminated from study participation.

The subpopulations analysed here include patients who were biologic-naïve, defined as patients with CD or UC who had not been previously treated with a biologic, and patients with a history of biologic failure, defined as patients with CD or UC who did not respond to, lost response to, or were intolerant to prior biologic agents.

### 2.2. Safety outcomes

Adverse events [AEs], SAEs, infections, and serious infections were coded according to the Medical Dictionary for Regulatory Activities [MedDRA version 24.1], and classified by system organ class, and preferred term [PT]. Other events of interest included: malignancies, major adverse cardiac events [MACE; cardiovascular death, non-fatal myocardial infarction, non-fatal stroke] [methods previously published],^6^ anaphylactic and serum sickness-like reactions, posterior reversible encephalopathy syndrome (PRES, also known as reversible posterior leukoencephalopathy syndrome [RPLS]), and opportunistic infections [OIs], including active tuberculosis [TB]. AEs were systematically collected in each trial throughout the study duration until the last study visit.

Infections were identified by the investigator on the case report form. To classify other infection events, the following categories were also analysed: *Clostridium difficile* (MedDRA PTs in the MedDRA higher-level term [HLT]: Clostridial infections); herpes zoster [PTs in the HLT: Herpes viral infections that also contain the words ‘zoster’ or ‘varicella’]; gastrointestinal [GI]-related abscesses that include the subcategories of anal, rectal, and perirectal infections [PTs: Anal abscess, Rectal abscess, Perirectal abscess]; abdominal and intestinal infections [PTs: Abdominal abscess, Abscess intestinal, Abdominal wall abscess, Colonic abscess]; and other abscesses [PTs: Groin abscess, Vaginal stoma site abscess, Vulval abscess, Pelvic abscess, Perineal abscess, and Genital abscess].

OIs, including active TB, were identified through sponsor clinical review guided by consensus recommendations in Winthrop *et al*.^8^ Events of disseminated herpes zoster are classified as OIs while all cases of herpes zoster are presented under other infection events.

For hypersensitivity reactions, the PTs Anaphylactic reaction, Anaphylactic shock, Anaphylactoid reaction, Anaphylactoid shock, Type I hypersensitivity, and Serum sickness or serum sickness-like reaction were included in the analysis.

As previously described,^6^ antibodies to ustekinumab were detected using a validated, drug-tolerant, electrochemiluminescence method on the Meso Scale Discovery® [MSD] platform in all phase 3 studies. In the phase 2 T07 and CERTIFI studies, an older, non-drug-tolerant validated bridging enzyme immunoassay was used. Of note, while the same MSD assay was used in all phase 3 studies, a slightly more stringent cut point was employed in the UC studies, as per updated regulatory guidelines. The original assay used a 5% false positive rate [FPR] for the screening assay and a nominal 0.1% [predicted 0.8%] FPR for the specificity assay; the updated assay used a 90% confidence interval [CI] for at least a 5% FPR for the screening assay and a nominal 1% [predicted 1.6%] FPR for the specificity assay. In all studies, a patient was considered positive for antibodies if treatment-emergent antibodies to ustekinumab were detected in the sample at any time, regardless of subsequent negative results.

### 2.3. Data analysis

Safety outcomes are presented as the number of events per 100 patient-years [PYs] of follow-up, or the number of patients per 100 PYs when indicated. This adjusts for potential differences in exposure between ustekinumab and placebo groups [because placebo patients either crossed over to receive ustekinumab or discontinued after study unblinding]. The 95% CIs are calculated based on an exact method assuming the observed number of events follows a Poisson distribution. All patients who received ≥1 dose of ustekinumab were included in the analysis of data through up to 5 years in CD and up to 4 years in UC.

Events were classified according to the actual treatment patients received during the study. Events were included in the placebo group for patients receiving placebo up until the time of administration of the first ustekinumab dose, or for ustekinumab-induced patients re-randomized to placebo, starting at 16 weeks following the ustekinumab induction [after >5 half-lives].

For analyses by dose [i.e. ustekinumab q8w or q12w], analyses included patients who received at least one dose of IV ustekinumab and one dose of ustekinumab 90 mg SC. Patients who only received a single 90 mg SC dose after an IV ustekinumab induction were counted to q8w or q12w dose according to the treatment assignment of the maintenance study, or counted to q8w dose if not assigned in the study. Patients who dose-adjusted from ustekinumab q12w to q8w were included in the q12w group. Patients from the T07 study were not included in the analysis due to study design—no patients received at least one ustekinumab IV and one ustekinumab SC dose.

Hazard ratios [ustekinumab:placebo] and corresponding 95% CIs from time-to-event analyses for SAEs and serious infections were based on a Cox proportional hazards model, with treatment as the explanatory variable, stratified by IBD study subtype [UC or CD], biologic failure status at baseline [Yes or No], and adjusting for age on entry into the trial [continuous], sex [F or M], ethnicity, and duration of disease [continuous variable defined from initial disease diagnosis to baseline visit in years]. For time-to-event analyses, Kaplan–Meier curves are presented, and a log-rank test was used to compare the proportion of people surviving from SAEs and serious infections between ustekinumab and placebo groups. In these analyses, times to first event were censored at 2 years, corresponding to the average duration patients were receiving placebo before being unblinded and terminated from study participation per protocol.

To assess baseline predictors to risk of SAEs and serious infections, a Cox regression was applied on six baseline characteristics based on previous analyses^9^: biologic failure status prior to study [Yes or No], corticosteroid use at baseline [Yes or No], sex [F or M], age [years], disease duration [years] prior to study, and baseline Crohn’s Disease Activity Index [CDAI] score^10^ or baseline Mayo score^11^ depending on the indication of the study [CD or UC, respectively].

For malignancies, comparisons with an external database were conducted to determine whether event rates were consistent with those expected in the general population. The expected number of patients with malignancies (other than non-melanoma skin cancer [NMSC] and cervical cancer *in situ*) is reported based on the external National Cancer Institute Surveillance, Epidemiology, and End Results [SEER] database [2019; https://seer.cancer.gov/resources^12^], adjusted for age, gender, and race. Standardized incidence ratios [SIRs] were calculated by dividing the observed number of patients with one or more malignancy in the pooled IBD population by the expected number of patients from SEER with one or more malignancy. Confidence intervals were calculated based on an exact method assuming that the observed number of patients with event follows a Poisson distribution.

## 3. Results

### 3.1. Baseline demographics and ustekinumab exposure

Through 5 years in the CD studies and 4 years in the UC studies, 1389 patients received placebo with 943 years of follow-up and 2575 patients received ustekinumab with 4826 years of follow-up [Supplementary Figure 1]. Approximately one-third of the IBD patients were exposed to ustekinumab for at least 3 years. Disease characteristics of the overall population at baseline [week 0 of induction] were described previously.^6^ Among patients in the pooled IBD population, 45.1% had a history of biologic failure and 23.0% were biologic-naïve; baseline demographics for biologic-naïve and biologic-failure subgroups are presented in Supplementary Table 1. Baseline characteristics were generally similar within the subgroups. At baseline [week 0 of induction], 49.7% of biologic failure and 43.5% of biologic-naïve IBD patients were receiving corticosteroids, and 28.5% of biologic failure and 32.6% of biologic-naïve IBD patients were receiving immunomodulators.

### 3.2. Adverse events

**Figure 1.**
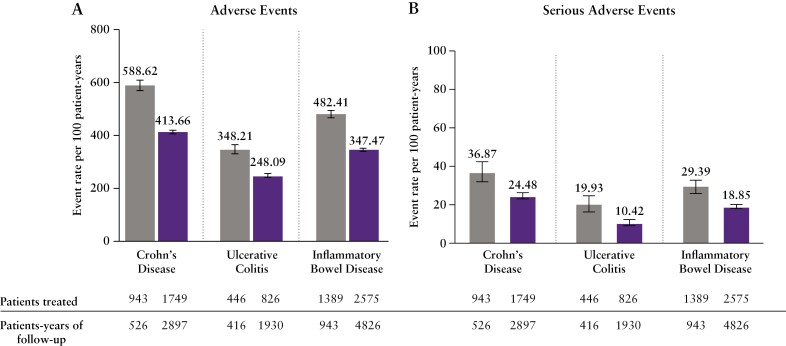
Rates of adverse events (A) and serious adverse events (B).

Rates [95% CI] of AEs were 482.41 [468.49, 496.64] per 100 PYs in the placebo group vs 347.47 [342.23, 352.77] per 100 PYs in the ustekinumab group across IBD [Figure 1A]. The most frequently reported AEs [event rate ≥7.00, excluding diseases under study] were headache, pyrexia, arthralgia, nausea, abdominal pain, upper respiratory tract infection [URTI], and nasopharyngitis; rates of these events were similar between ustekinumab and placebo treatment groups [Supplementary Figure 2A].

SAEs occurred at a rate of 29.39 [26.03, 33.06] per 100 PYs in the placebo group and 18.85 [17.65, 20.12] per 100 PYs in the ustekinumab group across IBD [Figure 1B]. The most frequently reported SAEs [event rate ≥0.40, excluding the diseases under study] were anaemia, anal abscess, constipation, intestinal obstruction, abdominal pain, and small intestinal obstruction; rates were similar between ustekinumab and placebo groups [Supplementary Figure 2B].

A surrogate marker for efficacy, rates for the AE PTs Crohn’s disease and Ulcerative colitis, were 21.86 vs 11.67 and 19.10 vs 6.44 [placebo vs ustekinumab], and rates for SAEs were 6.90 vs 4.08 and 3.50 vs 0.89, respectively, indicating that efficacious treatment^4,5^ reduces AEs related to the disease under study. Another surrogate marker of efficacy, rates of discontinuation due to AEs in the pooled IBD group were 11.56 [95% CI: 9.50, 13.95] for placebo and 5.51 [95% CI: 4.87, 6.22] for ustekinumab [Table 1].

**Table 1.** Key safety events for the overall population

	Crohn’s disease		Ulcerative colitis		Inflammatory bowel disease	
	Placebo^a^	Ustekinumab^b^	Placebo^a^	Ustekinumab^b^	Placebo^a^	Ustekinumab^b^
Patients treated	943	1749	446	826	1389	2575
Key safety events, rate per 100 PYs [*N*]						
MACE^c^	0.19 [1]	0.28 [8]	0.48 [2]	0.21 [4]	0.32 [3]	0.25 [12]
95% CI^d^	[0.00, 1.06]	[0.12, 0.54]	[0.06, 1.73]	[0.06, 0.53]	[0.07, 0.93]	[0.13, 0.43]
Discontinuation due to adverse event	11.78 [62]	6.87 [199]	11.29 [47]	3.47 [67]	11.56 [109]	5.51 [266]
95% CI^d^	[9.03, 15.11]	[5.95, 7.89]	[8.29, 15.01]	[2.69, 4.41]	[9.50, 13.95]	[4.87, 6.22]
Death	0.00 [0]	0.21 [6]	0.00 [0]	0.16 [3]	0.00 [0]	0.19 [9]
95% CI^d^	[0.00, 0.57]	[0.08, 0.45]	[0.00, 0.72]	[0.03, 0.45]	[0.00, 0.32]	[0.09, 0.35]

CI, confidence interval; MACE, major adverse cardiovascular event; PYs, patient years.

^a^Ulcerative colitis and Crohn’s disease: includes data up to the first ustekinumab dose for patients who were initially treated with placebo; includes data at or after 16 weeks from the first ustekinumab dose onward, up to the dose adjustment if patients had a dose adjustment, for patients who were crossed over or re-randomized to placebo maintenance.

^b^Ulcerative colitis and Crohn’s disease: includes data up to 16 weeks from the first ustekinumab dose for patients who were crossed over or re-randomized to placebo, and from the dose adjustment onward if had a dose adjustment from subcutaneous placebo to subcutaneous ustekinumab 90 mg q8w.

^c^For the ulcerative colits indication, MACE events were identified by clinical review and were not independently adjudicated.

^d^Confidence intervals based on an exact method assuming that the observed number of events follows a Poisson distribution.

**Figure 2.**
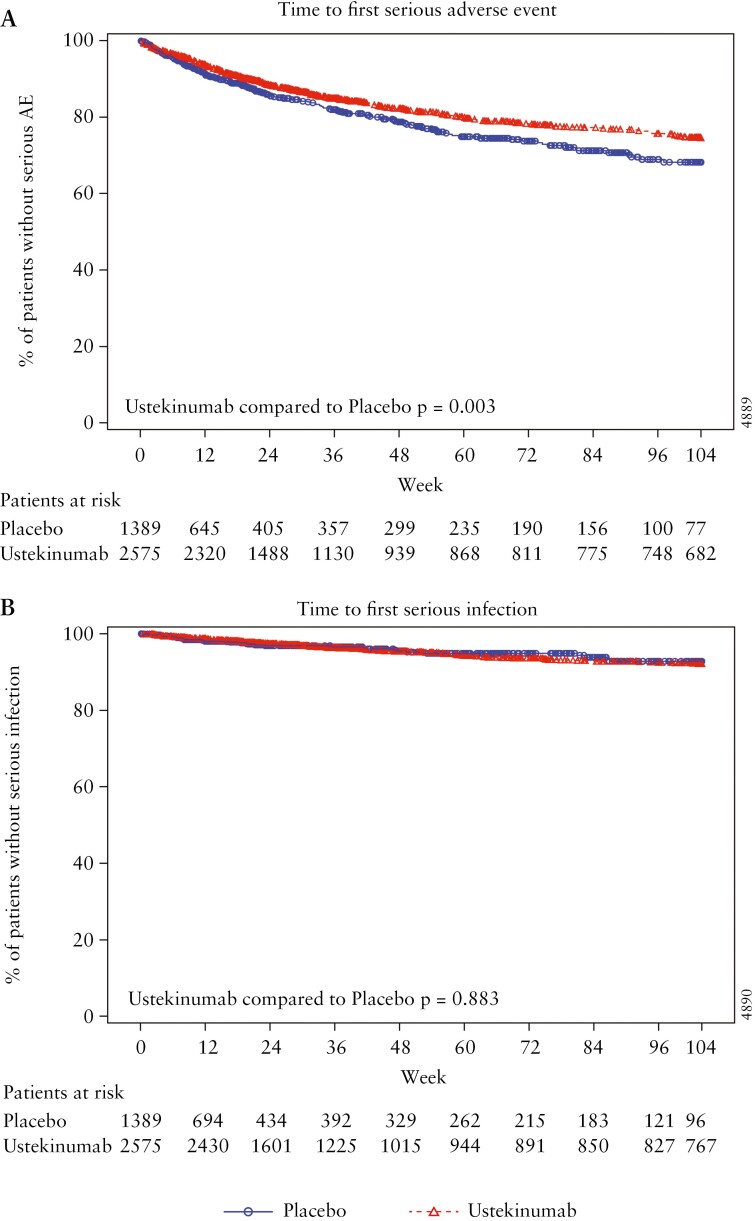
Time to first event analysis for serious adverse events (A) and serious infections (B).

Time to event analysis for ustekinumab showed statistically significant higher survival free of first serious event [*p* < 0.05; Figure 2A] and lower risk [hazard ratio with 95% CI, 0.732 [0.615, 0.870]) for SAEs. SAE rates in CD and UC were approximately two times higher in the placebo group compared with the ustekinumab group [Figure 1B], presumably driving this time-to-event analysis favouring ustekinumab.

Two baseline characteristic predictors for ustekinumab-treated patients with SAEs in CD were significant [*p* < 0.05]: prior biologic failure status and baseline CDAI score [Table 2]. For UC, significant [*p* < 0.05] predictors for ustekinumab-treated patients with SAEs were prior biologic failure status and corticosteroid use at baseline [Table 2].

**Table 2.** Predictors^a^ of serious adverse events

Risk factors	Adjusted hazard ratios [95% CI]	*p* value
**Crohn’s disease**		
Age	0.996 [0.987, 1.004]	0.3389
Sex: female	1.066 [0.886, 1.283]	0.4984
Biofailure status: No	0.604 [0.489, 0.746]	<0.0001
Disease duration, years	1.008 [0.997, 1.021]	0.1655
Baseline CDAI	1.003 [1.002, 1.005]	<0.0001
Baseline corticosteroid use: No	0.918 [0.764, 1.102]	0.3576
**Ulcerative colitis**		
Age	1.002 [0.990, 1.015]	0.7079
Sex: female	1.071 [0.773, 1.483]	0.6816
Biofailure status: No	0.611 [0.438, 0.852]	0.0037
Disease duration, years	1.006 [0.984, 1.029]	0.5833
Baseline Mayo score	1.010 [0.910, 1.120]	0.8516
Baseline corticosteroid use: No	0.684 [0.494, 0.948]	0.0224

CDAI, Crohn’s Disease Activity Index.

^a^Predictors for patients treated with ustekinumab.

Although study participants were required to adhere to appropriate birth control measures per study protocols, pregnancies were reported during the trials and data on pregnancy outcomes were requested for women who became pregnant. Consistent with previously reported results through 5 years of treatment in CD and 2 years in UC^13^ where ustekinumab treatment was not associated with a risk of negative outcomes during pregnancy, no additional congenital anomalies were observed among live births through the end of 4 years in UC.

### 3.3. Infections

**Figure 3.**
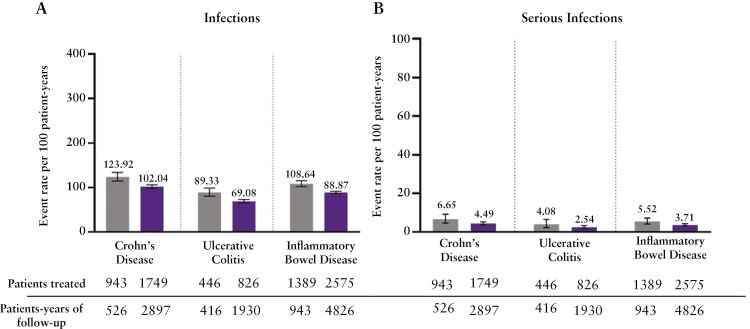
Rates of infections (A) and serious infections (B).

Infections were reported at a rate of 108.64 [95% CI:102.09, 115.50] per 100 PYs in the placebo group and 88.87 [86.23, 91.57] per 100 PYs in the ustekinumab group [Figure 3A]. The most frequently reported [event rate ≥2.00] infections with similar rates in the ustekinumab and placebo groups were nasopharyngitis, URTI, sinusitis, influenza, urinary tract infection, gastroenteritis, bronchitis, and oral herpes. [Supplementary Figure 3A].

Other infection events analysed were *C. difficile*; herpes zoster; GI-related abscesses that include the subcategories of anal, rectal, and perirectal infections; abdominal and intestinal infections; and other abscesses [Table 3]. For pooled IBD, rates for *C. difficile*, herpes zoster, and GI-related abscesses were no higher in the ustekinumab group compared to placebo.

**Table 3.** Other reported infections

	Crohn’s disease		Ulcerative colitis		Inflammatory bowel disease	
	Placebo^a^	Ustekinumab^b^	Placebo^a^	Ustekinumab^b^	Placebo^a^	Ustekinumab^b^
Patients treated	943	1749	446	826	1389	2575
Average follow-up [weeks]	29.01	86.13	48.55	121.47	35.29	97.46
Patient-years (PYs) of follow-up	526	2897	416	1930	943	4826
Number of patients with infections of interests per 100 PYs [number of patients with events]	10.07 [53]	3.97 [115]	2.88 [12]	1.45 [28]	6.90 [65]	2.96 [143]
*Clostridium difficile* infection	2.09 [11]	0.83 [24]	1.20 [5]	0.36 [7]	1.70 [16]	0.64 [31]
95% CI	[1.04, 3.74]	[0.53, 1.23]	[0.39, 2.80]	[0.15, 0.75]	[0.97, 2.76]	[0.44, 0.91]
Herpes zoster	1.71 [9]	0.86 [25]	0.72 [3]	0.83 [16]	1.27 [12]	0.85 [41]
95% CI	[0.78, 3.25]	[0.56, 1.27]	[0.15, 2.11]	[0.47, 1.35]	[0.66, 2.22]	[0.61, 1.15]
GI-related abscess	6.46 [34]	2.35 [68]	0.96 [4]	0.26 [5]	4.03 [38]	1.51 [73]
95% CI	[4.48, 9.03]	[1.82, 2.98]	[0.26, 2.46]	[0.08, 0.60]	[2.85, 5.53]	[1.19, 1.90]
Anal, rectal, and perirectal	5.13 [27]	1.52 [44]	0.72 [3]	0.21 [4]	3.18 [30]	0.99 [48]
95% CI	[3.38, 7.47]	[1.10, 2.04]	[0.15, 2.11]	[0.06, 0.53]	[2.15, 4.54]	[0.73, 1.32]
Abdominal and intestinal	0.95 [5]	0.28 [8]	0.00 [0]	0.00 [0]	0.53 [5]	0.17 [8]
95% CI	[0.31, 2.22]	[0.12, 0.54]	[0.00, 0.72]	[0.00, 0.16]	[0.17, 1.24]	[0.07, 0.33]
Abscess, other	0.38 [2]	0.59 [17]	0.24 [1]	0.10 [2]	0.32 [3]	0.39 [19]
95% CI	[0.05, 1.37]	[0.34, 0.94]	[0.01, 1.34]	[0.01, 0.37]	[0.07, 0.93]	[0.24, 0.61]

^a^Ulcerative colitis and Crohn’s disease: includes data up to the first ustekinumab dose for patients who were initially treated with placebo; includes data at or after 16 weeks from the first ustekinumab dose onward, up to the dose adjustment if patients had a dose adjustment, for patients who were crossed over or re-randomized to placebo maintenance.

^b^Ulcerative colitis and Crohn’s disease: includes data up to 16 weeks from the first ustekinumab dose for patients who were crossed over or re-randomized to placebo, and from the dose adjustment onward if patients had a dose adjustment from placebo SC to ustekinumab 90 mg SC q8w.

Overall, serious infections occurred at rates of 5.52 [95% CIs 4.12, 7.23] per 100 PYs in the placebo group and 3.71 [3.19, 4.29] per 100 PYs in the ustekinumab group [Figure 3B]. The most frequently reported serious infections were anal abscess, pneumonia, cellulitis, abdominal abscess, *C. difficile* colitis, postoperative abscess, rectal abscess, liver abscess, pneumonia viral, and gastroenteritis [Supplementary Figure 3B].

Time to event analysis also showed similar survival free of and risk of time to first serious infection [Figure 2B], i.e. no higher rate of serious infection with ustekinumab treatment [*p* = 0.883], and no difference in risk (hazard ratio with 95% CI: 0.928 [0.646, 1.334]) for serious infections compared to placebo.

There were no significant predictors [*p* < 0.05] of serious infection for patients in the ustekinumab group in either CD or UC among the variables assessed [Supplementary Table 2].

### 3.4. Opportunistic infections including TB

**Figure 4.**
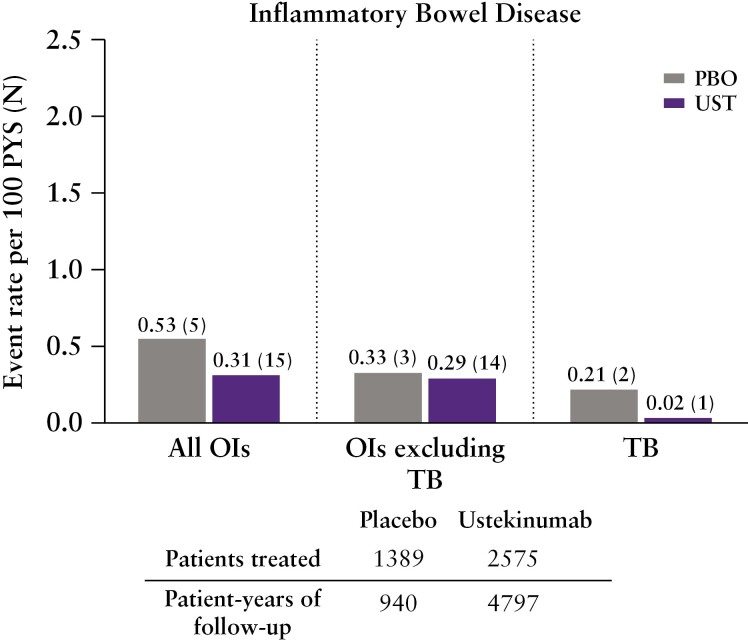
Opportunistic infections. OIs, opportunistic infections; TB, tuberculosis.

Overall, OIs including active TB in ustekinumab-treated patients were reported infrequently. In total, there were 20 patients with OIs in IBD, including 15 ustekinumab-treated patients and five placebo-treated patients [Figure 4]. Overall rates of OIs were 0.31 for ustekinumab-treated patients and 0.53 for placebo-treated patients. The most frequently reported OIs were oesophageal candidiasis and cytomegalovirus. A full list of OIs [excluding TB] is presented in Supplementary Table 3. Through up to 5 years of follow-up, 16 of the 17 patients with OIs [excluding TB] were receiving concomitant immunosuppressants [including corticosteroids].

Three active TB cases [all previously described] were reported in IBD patients: one in the ustekinumab group [0.02 per 100 PYs] and two in the placebo group [0.21 per 100 PYs]. The single case in the ustekinumab group occurred in an asymptomatic 45-year-old South African CD patient treated with ustekinumab q8w during the long-term extension, who had a positive QuantiFERON®-TB Gold test on routine screening and bronchial brushings positive for *Mycobacterium tuberculosis* [patient was receiving concomitant steroids].^14^ In the placebo group, one case was reported in a 32-year-old Hungarian CD patient, 10 months after receiving their last ustekinumab dose [patient was receiving concomitant steroids]^4^; the second case was in a 52-year-old Korean UC patient with pulmonary TB who received placebo and never received ustekinumab in the 1 year maintenance period.^6^

### 3.5. Key safety events in subgroups

The analysis of key safety by ustekinumab maintenance doses [Supplementary Table 4], q8w or q12w, included 1344 patients treated with placebo, 461 patients treated with ustekinumab q12w, and 1304 patients treated with ustekinumab q8w, with 934, 1559, and 2997 PYs of follow-up, respectively. Of note, differences in the number of patients in each group occur due to study designs and ensuring the inclusion of patients treated with one or more SC ustekinumab maintenance dose after ustekinumab IV induction. Overall, rates of key safety events in UC, CD, or pooled IBD were no higher in the ustekinumab q8w and q12w groups than the placebo group, with no apparent dose effect observed.

Rates of all key safety events in the IBD group were no higher in the ustekinumab group than in the placebo group for both patients who were biologic naïve [Supplementary Table 5] and patients who had a history of prior biologic failure [Supplementary Table 6].

### 3.6. Hypersensitivity reactions

No serious anaphylactic reactions or serum sickness-like reactions due to ustekinumab were reported through 5 years in CD and through 4 years in UC. As previously reported, in the US Prescribing Information,^1^ two non-serious cases of hypersensitivity reactions occurred in CD patients. One case of signs/symptoms of hypersensitivity [tightness of the throat, shortness of breath, and flushing] temporally associated with treatment occurred in one patient after SC injection and one in a patient after IV infusion. Symptoms resolved within 1 hour after oral corticosteroid/antihistamine treatment.

Through 5 years of CD and 4 years of UC treatment, antibodies to ustekinumab remained low. For CD patients, 5.8% of patients treated with continuous ustekinumab from induction through the final safety visit of the long-term maintenance study were positive for antibodies to ustekinumab. Similarly, 5.5% of UC patients who received ustekinumab in the maintenance study and remained on ustekinumab in the long-term extension were positive for antibodies to ustekinumab.

### 3.7. Malignancies

**Figure 5.**
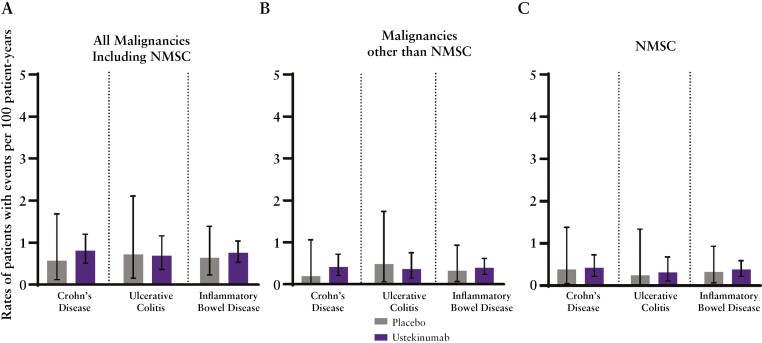
Rates of all malignancies including non-melanoma skin cancer (NMSC) (A), malignancies other than NMSC (B), and NMSC (C).

No events of lymphoma were reported through 5 years of treatment in CD and 4 years of treatment in UC. Overall rates of all malignancies including NMSC were similar between the ustekinumab and placebo groups [Figure 5A]. Rates of malignancies other than NMSC for the pooled IBD group were 0.32 [95% CI: 0.07, 0.93] patients per 100 PYs for the placebo group and 0.39 [0.24, 0.62] per 100 PYs for the ustekinumab group [Figure 5B]. The rate of NMSC was 0.32 [0.07, 0.93] per 100 PYs for the placebo group and 0.38 [0.22, 0.59] per 100 PYs for the ustekinumab group [Figure 5C]. The most frequently reported malignancies (>2 patients; rates [patients with specified events]) were basal cell carcinoma (placebo vs ustekinumab; 0.14 [2] vs 0.54 [14]), squamous cell carcinoma [SCC] (0.14 [2] vs 0.23[6]), and melanoma (0.07 [1] vs 0.11 [3]). Malignancies reported [excluding NMSC] for the combined IBD group [in either the placebo or ustekinumab treatment group] were melanoma, prostate, renal, rectal, and small intestine. Types of malignancies other than NMSC are listed by treatment group in Supplementary Table 7.

**Figure 6.**
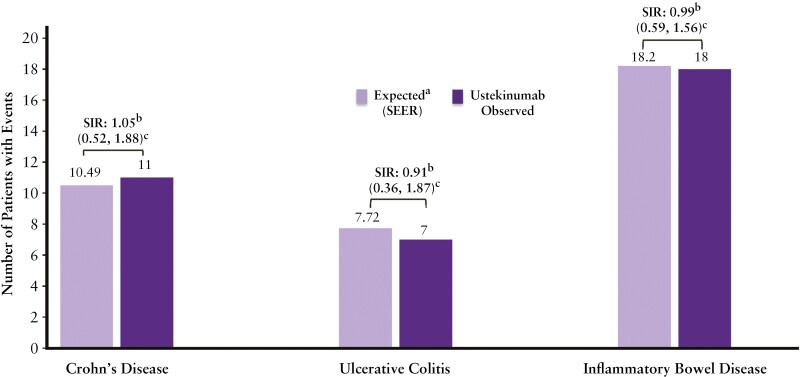
Observed rates of malignancies compared with expected (SEER) and standard incidence rations (SIR). ^a^The expected number of patients with malignancies is based on the National Cancer Institute Surveillance, Epidemiology, and End Results (SEER) database (year 2019), adjusted for age, gender, and race. Only patients with race belonging to White, Black or African American, Asian, American Indian/Alaska Native, Native Hawaiian, or other Pacific Islander were included as SEER only contains incidence rates for these populations. One malignancy was not included as the patient’s race was unknown. ^b^[SIR]; observed number of patients with malignancy divided by expected number of patients with malignancy. ^c^Confidence intervals based on an exact method assuming that the observed number of events follows a Poisson distribution.

Comparing results from pooled long-term safety with SEER, the SIR [95% CI] for malignancies [other than NMSC and cervical cancer *in situ*] in the ustekinumab group for CD, UC, and all IBD patients was ~1, indicating no increased risk of malignancy with ustekinumab treatment [Figure 6].

### 3.8. Major adverse cardiovascular events and deep vein thrombosis/pulmonary embolism

MACE occurred infrequently and at similar rates between treatment groups: 0.32 [95% CI: 0.07, 0.93] per 100 PYs in the placebo-treated patients and 0.25 [0.13, 0.43] per 100 PYs in ustekinumab-treated patients in the combined IBD group [Table 1]. Component rates for myocardial infarction or stroke across IBD were similar in the ustekinumab group compared with the placebo group. Each of the patients with events presented with confounding comorbidities at the time of the events.

Analyses for deep vein thrombosis/pulmonary embolism [DVT/PE] are presented as rates of events per 100 PYs. Overall, across IBD, events of DVT and PE were infrequently reported through 5 years in CD and 4 years in UC. Rates for the PT DVT were 0.11 [95% CI: 0.00, 0.59] vs 0.35 [0.21, 0.56] for the placebo and ustekinumab groups, respectively, and rates for the PT PE were 0.42 [0.12, 1.09] vs 0.08 [0.02, 0.21], respectively. Overall rates of DVT and PE were 0.53 [0.17, 1.24] for placebo vs 0.44 [0.27, 0.67] for ustekinumab.

### 3.9. Neurological events

No cases of PRES [previously referred to as RPLS] were reported through 5 years of CD and 4 years of UC.

### 3.10. Deaths

For the pooled IBD population, there were three deaths through the end of the UC studies and six though the end of the CD studies. All deaths were considered unrelated to study agent. Details of all deaths can be found in Supplementary Table 8.

## 4. Discussion

The long-term safety data presented among 2575 patients treated with ustekinumab with 4826 PYs of follow-up in this analysis support the well-established safety profile of ustekinumab across IBD indications. Previously published safety data through 1 year in CD and UC showed a favourable safety profile for ustekinumab compared to placebo, and the long-term data presented here through 5 years in CD and 4 years in UC provided further reassurance of the safety of ustekinumab in IBD.

In this analysis, rates of key safety events, including MACE and malignancies, were similar between placebo and ustekinumab, or not higher for ustekinumab. OIs, including TB, were reported infrequently. One case of disseminated histoplasmosis [previously described from the T07 CD study]^6^ was reported in a patient who received a single 4.5 mg/kg IV dose of ustekinumab after discontinuing infliximab therapy 3 months previously. There were no additional histoplasmosis cases in the subsequent Phase 2 and 3 IBD studies. Key safety parameters in patients who were biologic-naïve and those with prior biologic failure showed that rates in the IBD group were no higher in the ustekinumab group than in the placebo group for each subgroup. Additionally, no lymphomas or cases of PRES [previously referred to as RPLS] were reported. Rates of NMSC were similar between the ustekinumab and placebo groups. Through 5 years in CD and 4 years in UC, nine deaths were reported in ustekinumab-treated patients, six in CD and three in UC; all were considered to be unrelated to the study agent.

To add to the previously presented safety data, this analysis allowed a look into the long-term safety of ustekinumab by maintenance SC dose [q8w and q12w]. These data from the long-term analysis did not demonstrate a dose response in key safety parameters. Although no difference was observed between ustekinumab SC dose [q8w or q12w] and placebo, the overall AE and infection rates were numerically higher in the SC q8w group than the SC q12w group. This was influenced by the analysis rules which took patients who received an IV and a single SC ustekinumab dose in the CERTIFI and UNIFI induction studies and placed them all into the q8w group [non-randomized], making this group larger than would have been assigned by study design. The data from such patients may bias the q8w treatment group. The q12w group was smaller and included only those patients who were assigned to the q12w regimen in IM-UNITI and the UNIFI maintenance study.

One limitation of this study is the longer-term follow-up for ustekinumab vs placebo, as longer follow-up cannot be obtained with placebo even when results are presented as rates adjusted with time. In the absence of a long-term placebo comparator for the longer studies, which would not be an ethical study design, the unblinding only after the last patient in the randomized maintenance phase has completed follow-up gives valuable placebo comparator data.

An important consideration in the treatment of IBD is that safety is dependent on efficacy, and untreated or undertreated disease should be considered an AE. This concept is further supported by surrogate makers in this data set, rates of AEs for the disease under study [CD or UC], and events leading to discontinuation of study treatment. Event rates in patients treated with ustekinumab were lower and approximately half of rates for patients treated with placebo in these surrogate efficacy markers, demonstrating that ustekinumab treats the disease under study resulting in fewer CD and UC events in the ustekinumab group. Additional evidence was also presented in the time-to-event analyses showing that there were statistically significantly fewer ustekinumab-treated patients reporting serious events. Further supporting the concept that untreated disease is an AE, the most frequently reported SAEs in this analysis were the diseases under study or symptoms related to the disease, which were reported at higher rates in the placebo group.

In a recent publication,^15^ the safety of ustekinumab was compared to thiopurine/anti-tumour necrosis factor [TNF] combination treatment, anti-TNF monotherapy, tofacitinib, and vedolizumab. As noted by the authors, risks of infections, malignancies, immunogenicity, and metabolic disorders may vary based on treatment options and should be considered along with their efficacy during patient/physician decision-making. In this long-term safety analysis, no increased risk of serious infection or OIs [including TB] was identified. In addition, time-to-event analysis showed no difference in rates of serious infections between ustekinumab and placebo treatment, consistent with a published multicentre cohort of patients treated with biologics, in which ustekinumab was associated with a lower risk of serious infections compared with TNF-α antagonists and vedolizumab.^16^

Overall, the 4826 PYs of follow up in this analysis through the end of the long-term safety follow-up in CD and UC studies demonstrated that overall rates of key safety events were similar between treatment groups or no higher for ustekinumab. No new safety concerns were identified through up to 5 years in CD and 4 years of treatment in UC. This final compilation of cumulative ustekinumab safety data through 5 years in CD and 4 years in UC continues to support the well-established safety profile of ustekinumab in IBD and across all approved indications.

## Supplementary Data

Supplementary data are available online at *ECCO-JCC* online.

jjae013_suppl_Supplementary_Figures_1-3_Tables_1-8

## Data Availability

The data sharing policy of Janssen Pharmaceutical Companies of Johnson & Johnson is available at https://www.janssen.com/clinical-trials/transparency. As noted on this site, requests for access to the study data can be submitted through Yale Open Data Access [YODA] Project site at http://yoda.yale.edu.
