# Supplementary material for: Safety of Ustekinumab in Inflammatory Bowel Disease: Pooled Safety Analysis Through 5 Years in Crohn’s Disease and 4 Years in Ulcerative Colitis
Source: J Crohns Colitis. 2024 Feb 4;18(7):1091–101. doi: 10.1093/ecco-jcc/jjae013 (PMC11302965; doi:10.1093/ecco-jcc/jjae013)
Supplement: jjae013_suppl_Supplementary_Figures_1-3_Tables_1-8 [file jjae013_suppl_supplementary_figures_1-3_tables_1-8.docx]

***Supplemental Tables and Figures***

**Supplemental Table 1. Baseline Demographics for Patients who are Biologic-naïve or with a History of Biologic Failure**

|  | Crohn’s Disease | Ulcerative Colitis | Inflammatory Bowel Disease |
| --- | --- | --- | --- |
| ***Biologic Failure*** | | | |
| Patients Treated | 1298 | 491 | 1789 |
| Age (years) |  |  |  |
| Mean (SD) | 38.1 (12.33) | 41.9 (13.95) | 39.1 (12.90) |
| Sex |  |  |  |
| Male | 548 (42.2%) | 300 (61.1%) | 848 (47.4%) |
| Female | 750 (57.8%) | 191 (38.9%) | 941 (52.6%) |
| Race |  |  |  |
| White | 1141 (87.9%) | 332 (67.6%) | 1473 (82.3%) |
| Black or African American | 42 (3.2%) | 9 (1.8%) | 51 (2.9%) |
| Asian | 73 (5.6%) | 89 (18.1%) | 162 (9.1%) |
| American Indian or Alaska Native | 0 | 0 | 0 |
| Native Hawaiian or other Pacific Islander | 0 | 0 | 0 |
| Other | 24 (1.8%) | 17 (3.5%) | 41 (2.3%) |
| Unknown | 2 (0.2%) | 3 (0.6%) | 5 (0.3%) |
| Not Reported | 16 (1.2%) | 41 (8.4%) | 57 (3.2%) |
| Body Mass Index (kg/m^2^) |  |  |  |
| Mean (SD) | 24.73 (6.116) | 24.71 (4.751) | 24.72 (5.772) |
| Age at disease diagnosis (years) |  |  |  |
| Mean (SD) | 25.8 (11.69) | 32.7 (14.13) | 27.7 (12.78) |
| Duration of disease (years) |  |  |  |
| Mean (SD) | 12.28 (8.647) | 9.24 (7.320) | 11.44 (8.412) |
| Concomitant therapies |  |  |  |
| Corticosteroid ^a^ | 616 (47.5%) | 273 (55.6%) | 889 (49.7%) |
| Immunomodulators | 369 (28.4%) | 140 (28.5%) | 509 (28.5%) |
| 6-MP/AZA | 252 (19.4%) | 135 (27.5%) | 387 (21.6%) |
| MTX | 122 (9.4%) | 5 (1.0%) | 127 (7.1%) |
| Corticosteroids^a^ and immunomodulators | 169 (13.0%) | 62 (12.6%) | 231 (12.9%) |
| Smoking status |  |  |  |
| Current smoking | 313 (24.1%) | 24 (4.9%) | 337 (18.8%) |
| ***Biologic Naive*** | | | |
| Patients treated | 469 | 442 | 911 |
| Age (years) |  |  |  |
| Mean (SD) | 39.2 (13.62) | 41.4 (13.31) | 40.3 (13.51) |
| Sex |  |  |  |
| Male | 224 (47.8%) | 268 (60.6%) | 492 (54.0%) |
| Female | 245 (52.2%) | 174 (39.4%) | 419 (46.0%) |
| Race |  |  |  |
| White | 405 (86.4%) | 380 (86.0%) | 785 (86.2%) |
| Black or African American | 14 (3.0%) | 0 | 14 (1.5%) |
| Asian | 33 (7.0%) | 47 (10.6%) | 80 (8.8%) |
| American Indian or Alaska Native | 0 | 0 | 0 |
| Native Hawaiian or other Pacific Islander | 1 (0.2%) | 0 | 1 (0.1%) |
| Other | 14 (3.0%) | 11 (2.5%) | 25 (2.7%) |
| Unknown | 1 (0.2%) | 0 | 1 (0.1%) |
| Not Reported | 1 (0.2%) | 4 (0.9%) | 5 (0.5%) |
| Body Mass Index (kg/m^2^) |  |  |  |
| Mean (SD) | 25.39 (6.113) | 24.76 (5.505) | 25.08 (5.831) |
| Age at disease diagnosis (years) |  |  |  |
| Mean (SD) | 31.1 (12.81) | 34.6 (13.19) | 32.8 (13.11) |
| Duration of disease (years) |  |  |  |
| Mean (SD) | 8.21 (9.061) | 6.77 (7.377) | 7.51 (8.314) |
| Concomitant therapies |  |  |  |
| Corticosteroid^a^ | 176 (37.5%) | 220 (49.8%) | 396 (43.5%) |
| Immunomodulators | 175 (37.3%) | 122 (27.6%) | 297 (32.6%) |
| 6-MP/AZA | 159 (33.9%) | 118 (26.7%) | 277 (30.4%) |
| MTX | 16 (3.4%) | 4 (0.9%) | 20 (2.2%) |
| Corticosteroids^a^ and immunomodulators | 48 (10.2%) | 55 (12.4%) | 103 (11.3%) |
| Smoking status |  |  |  |
| N | 469 | 442 | 911 |
| Current smoking | 124 (26.4%) | 21 (4.8%) | 145 (15.9%) |
| ^a^ Includes budesonide and beclomethasone dipropionate.  6-MP/AZA, 6-mercaptopurine/azathioprine; MTX, methotrexate; SD, standard deviation | | | |

**Supplemental Table 2.** Predictors^*^ of serious infections

| **Risk Factors** | **Adjusted Hazard Ratios (95% CI)** | ***P* value** |
| --- | --- | --- |
| ***Crohn’s disease*** | | |
| Age | 1.005 (0.990, 1.021) | 0.4948 |
| Sex: female | 0.932 (0.658, 1.321) | 0.6923 |
| Biofailure status: No | 0.688 (0.469, 1.010) | 0.0565 |
| Disease duration- yrs | 1.004 (0.983, 1.026) | 0.7109 |
| Baseline CDAI | 1.003 (1.000, 1.005) | 0.0822 |
| Baseline corticosteroid use- No | 0.970 (0.685, 1.372) | 0.8620 |
| ***Ulcerative Colitis*** | | |
| Age | 1.003 (0.980, 1.025) | 0.8239 |
| Sex: female | 1.573 (0.881, 2.809) | 0.1259 |
| Biofailure status: No | 0.663 (0.366, 1.203) | 0.1768 |
| Disease duration- yrs | 1.027 (0.989, 1.066) | 0.1619 |
| Baseline Mayo score | 0.985 (0.817, 1.187) | 0.8727 |
| Baseline corticosteroid use- No | 1.264 (0.704, 2.271) | 0.4325 |

^*^Predictors for patients treated with ustekinumab.

**Supplemental Table 3. Details for Opportunistic Infections (Excluding TB)**

| **OI Diagnosis** | **Age/Sex** | **Treatment** | **Details/ Concomitant medications** |  |
| --- | --- | --- | --- | --- |
| ***Crohn’s disease*** | | | |  |
| Oesophageal candidiasis | 39/M | Placebo | Corticosteroids | |
| Oesophageal candidiasis | 67/F | Placebo | Corticosteroids, methotrexate | |
| CMV colitis | 31/M | Placebo | None | |
| Disseminated histoplasmosis | 33/F | Ustekinumab | Fever 2 days prior to first ustekinumab dose; received infliximab 3 months prior; prednisone, azathioprine | |
| Oesophageal candidiasis | 54/F | Ustekinumab | Inhaled fluticasone propionate and mesalazine | |
| Oesophageal candidiasis | 36/M | Ustekinumab | Corticosteroids and oral budesonide | |
| Cryptosporidiosis infection | 28/F | Ustekinumab | Corticosteroids | |
| Meningitis listeria | 33/M | Ustekinumab | Corticosteroids; field worker | |
| Oesophageal candidiasis | 31/F | Ustekinumab | Infliximab 2 weeks after last ustekinumab and prior to OI diagnosis | |
| ***Ulcerative Colitis*** | | | |  |
| Pneumonia legionella | 49/F | Ustekinumab | Symptoms 3 weeks after last ustekinumab dose; infliximab and intravenous methylprednisolone 4 days prior to opportunistic infection diagnosis | |
| CMV colitis | 51/F | Ustekinumab | Corticosteroids | |
| CMV colitis | 31/M | Ustekinumab | Corticosteroids, azathioprine | |
| Ophthalmic herpes simples and oral herpes simplex | 29/M | Ustekinumab | Corticosteroids | |
| CMV colitis | 71/M | Ustekinumab | Corticosteroids, rectal mesalazine | |
| Listeriosis | 84/F | Ustekinumab | Prednisolone, azathioprine; opportunistic infection led to colectomy due to worsening of colitis, and complicated hospital stay including DIC, intubation, and multiple other infection | |
| CMV colitis | 27/M | Ustekinumab | Prednisone; CMV colitis complicated by pneumofibrosis, urolithiasis, hydrothorax. Treated with cytoflavin and other antimicrobials and improved. | |
| HSV mucositis and neutropenia | 19/F | Ustekinumab | 6-methylprednisone, prednisolone, and budesonide | |

CMV colitis; cytomegalovirus colitis; HSV, herpes simplex virus; TB, tuberculosis

**Supplemental Table 4**. **Key Safety Events by Ustekinumab SC Maintenance Dose (q8w and q12w)**

|  | **Crohn’s Disease** | | | **Ulcerative Colitis** | | | **Inflammatory Bowel Disease** | | |
| --- | --- | --- | --- | --- | --- | --- | --- | --- | --- |
|  | **Placebo^a^** | **Ustekinumab** | | **Placebo^a^** | **Ustekinumab** | | **Placebo^a^** | **Ustekinumab** | |
|  |  | **q12w^b^** | **q8w^b^** |  | **q12w^b^** | **q8w^b^** |  | **q12w^b^** | **q8w^b^** |
| Patients Treated | 898 | 289 | 839 | 446 | 172 | 465 | 1344 | 461 | 1304 |
| Average follow-up (weeks) | 29.94 | 178.89 | 104.99 | 48.55 | 170.71 | 145.76 | 36.12 | 175.84 | 119.53 |
| Patient-years of follow-up | 517 | 994 | 1694 | 416 | 565 | 1303 | 934 | 1559 | 2997 |
| Key safety events, Rate per 100 patient-years |  |  |  |  |  |  |  |  |  |
| Adverse Events | 578.82 | 347.71 | 411.58 | 348.21 | 211.99 | 253.02 | 475.95 | 298.55 | 342.63 |
| 95% CI | (558.26, 599.93) | (336.22, 359.50) | (401.98, 421.36) | (330.52, 366.61) | (200.15, 224.35) | (244.45, 261.80) | (462.05, 490.15) | (290.04, 307.26) | (336.03, 349.32) |
| Serious Adverse Events | 36.94 | 20.12 | 23.02 | 19.93 | 8.86 | 9.59 | 29.35 | 16.04 | 17.18 |
| 95% CI | (31.88, 42.56) | (17.42, 23.11) | (20.79, 25.43) | (15.88, 24.71) | (6.57, 11.67) | (7.98, 11.43) | (25.98, 33.04) | (14.11, 18.15) | (15.73, 18.73) |
| Infections | 122.80 | 96.76 | 101.66 | 89.33 | 63.76 | 69.35 | 107.87 | 84.81 | 87.61 |
| 95% CI | (113.44, 132.74) | (90.74, 103.07) | (96.91, 106.57) | (80.49, 98.89) | (57.34, 70.70) | (64.91, 74.03) | (101.31, 114.75) | (80.30, 89.50) | (84.29, 91.02) |
| Serious Infections | 6.58 | 5.13 | 3.31 | 3.60 | 2.66 | 1.76 | 5.25 | 4.23 | 2.64 |
| 95% CI | (4.55, 9.19) | (3.82, 6.74) | (2.50, 4.29) | (2.02, 5.94) | (1.49, 4.38) | (1.12, 2.65) | (3.88, 6.94) | (3.27, 5.39) | (2.09, 3.28) |

CI, confidence interval; q12w, every 12 weeks; q8w, every 8 weeks

^a^Includes data up to the first ustekinumab dose for patients who were initially treated with placebo; includes data at or after 16 weeks from the first ustekinumab dose onward, up to the dose adjustment if patients had a dose adjustment, for patients who were crossed over or rerandomized to placebo maintenance.
^b^Based on treatment group assigned in the individual study. Included patients who received at least one-dose of ustekinumab IV and one dose of ustekinumab 90 mg SC, with data up to 16 weeks from the first ustekinumab dose for patients who were crossed over or rerandomized to placebo, and from the dose adjustment onward if patients had a dose adjustment from placebo SC to ustekinumab 90 mg SC q8w.

**Supplemental Table 5. Key Safety Events in Patients who were Biologic Naive**

|  | **Crohn’s Disease** | | **Ulcerative Colitis** | | **Inflammatory Bowel Disease** | |
| --- | --- | --- | --- | --- | --- | --- |
|  | **Placebo^a^** | **Ustekinumab^b^** | **Placebo^a^** | **Ustekinumab^b^** | **Placebo^a^** | **Ustekinumab^cb^** |
| Patients Treated | 215 | 400 | 210 | 371 | 425 | 771 |
| Average follow-up (weeks) | 43.18 | 129.72 | 51.03 | 138.33 | 47.06 | 133.86 |
| Patient-years of follow-up | 179 | 998 | 206 | 987 | 385 | 1985 |
| Key safety events, Rate |  |  |  |  |  |  |
| Adverse Events | 439.09 | 278.91 | 254.27 | 190.70 | 340.07 | 235.04 |
| 95% CI | (408.89, 470.93) | (268.64, 289.47) | (232.97, 277.01) | (182.18, 199.51) | (321.89, 359.01) | (228.35, 241.89) |
| Serious Adverse Events | 20.16 | 12.33 | 11.65 | 7.60 | 15.60 | 9.98 |
| 95% CI | (14.12, 27.91) | (10.24, 14.71) | (7.46, 17.33) | (5.98, 9.53) | (11.90, 20.08) | (8.63, 11.47) |
| Infections | 106.41 | 73.86 | 55.80 | 49.65 | 79.30 | 61.82 |
| 95% CI | (91.82, 122.67) | (68.62, 79.39) | (46.07, 66.98) | (45.35, 54.25) | (70.65, 88.71) | (58.41, 65.38) |
| Serious Infections^c^ | 5.60 | 2.31 | 0.97 | 1.52 | 3.12 | 1.91 |
| 95% CI | (2.69, 10.30) | (1.46, 3.46) | (0.12, 3.51) | (0.85, 2.51) | (1.61, 5.45) | (1.35, 2.63) |

CI, confidence interval

^a^Ulcerative Colitis and Crohn's Disease: includes data up to the first ustekinumab dose for patients who were initially treated with placebo; includes data at or after 16 weeks from the first ustekinumab dose onward, up to the dose adjustment if patients had a dose adjustment, for patients who were crossed over or rerandomized to placebo maintenance.
^b^Ulcerative Colitis and Crohn's Disease: includes data up to 16 weeks from the first ustekinumab dose for patients who were crossed over or rerandomized to placebo, and from the dose adjustment onward if patients had a dose adjustment from placebo SC to ustekinumab 90 mg SC q8w.
^c^The events of serious infection were reviewed by clinical.

**Supplemental Table 6. Key Safety Events in Patients with a History of Biologic Failure**

|  | **Crohn’s Disease** | | **Ulcerative Colitis** | | **Inflammatory Bowel Disease** | |
| --- | --- | --- | --- | --- | --- | --- |
|  | **Placebo^a^** | **Ustekinumab^b^** | **Placebo^a^** | **Ustekinumab^b^** | **Placebo^a^** | **Ustekinumab^b^** |
| Patients Treated | 621 | 1164 | 226 | 432 | 847 | 1596 |
| Average follow-up (weeks) | 24.06 | 64.97 | 46.62 | 105.84 | 30.08 | 76.03 |
| Patient-years of follow-up | 287 | 1454 | 203 | 879 | 490 | 2334 |
| Key safety events, Rate |  |  |  |  |  |  |
| Adverse Events | 678.77 | 517.86 | 441.77 | 312.53 | 580.76 | 440.49 |
| 95% CI | (648.98, 709.58) | (506.23, 529.69) | (413.29, 471.68) | (300.95, 324.44) | (559.61, 602.50) | (432.02, 449.09) |
| Serious Adverse Events | 48.38 | 33.08 | 28.63 | 13.42 | 40.21 | 25.67 |
| 95% CI | (40.68, 57.13) | (30.19, 36.17) | (21.74, 37.01) | (11.11, 16.07) | (34.79, 46.24) | (23.65, 27.81) |
| Infections | 132.97 | 121.30 | 124.39 | 89.28 | 129.42 | 109.23 |
| 95% CI | (119.97, 147.00) | (115.70, 127.09) | (109.50, 140.73) | (83.14, 95.75) | (119.54, 139.90) | (105.03, 113.56) |
| Serious Infections | 7.66 | 5.64 | 6.42 | 2.96 | 7.14 | 4.63 |
| 95% CI | (4.80, 11.59) | (4.48, 7.00) | (3.42, 10.97) | (1.93, 4.33) | (4.98, 9.94) | (3.80, 5.59) |

CI, confidence

^a^Ulcerative Colitis and Crohn's Disease: includes data up to the first ustekinumab dose for patients who were initially treated with placebo; includes data at or after 16 weeks from the first ustekinumab dose onward, up to the dose adjustment if patients had a dose adjustment, for patients who were crossed over or rerandomized to placebo maintenance.
^b^ Ulcerative Colitis and Crohn's Disease: includes data up to 16 weeks from the first ustekinumab dose for patients who were crossed over or rerandomized to placebo, and from the dose adjustment onward if patients had a dose adjustment from placebo SC to ustekinumab 90 mg SC q8w.

**Supplemental Table 7. Types of Malignancy in IBD Group^*^ (Other than NMSC)**

| **Placebo** | **Ustekinumab (number of patients*)** |
| --- | --- |
| Melanoma (1) | Melanoma (3) |
| Testicular (1) | Testicular (1) |
| Thyroid (1) | Prostate (2) |
|  | Renal (2) |
|  | Plasma cell myeloma (1) |
|  | Endometrial (1) |
|  | Rectal (2) |
|  | Small intestine (2) |
|  | Colorectal (2) |
|  | CML (1) |
|  | Pancreatic (1) |
|  | Breast (1) |
|  |  |

IBD, inflammatory bowel disease; NMSC, non-melanoma skin cancer

^*^Types of malignancy occurring in 1 or more patients

**Supplemental Table 8. Deaths**

| **Cause** | **Age/Sex** | **Details** |
| --- | --- | --- |
| ***Crohn’s disease*** | | |
| Cardio-respiratory arrest | 56/M | Coronary heart disease and 2 additional cardiovascular risk factor, died after bowel surgery |
| Asphyxia (by hanging) | 24/M | Found dead by suicide after incarceration for driving intoxicated |
| Sudden death | 61/M | Found dead; presumed secondary to ventricular arrythmia; no autopsy |
| End stage renal disease | 72/F | Discontinued dialysis on study day 809 |
| Acute myocardial infarction | 46/M | 3 cardiovascular risk factors; 10 days after experiencing unstable angina and refusing ER; per autopsy cause of death acute heart failure |
| Septic shock | 33/F | Primary investigator discontinued patient 3 months prior; post-op subcutaneous supraumbilical seroma from hernia surgery; CT large haematoma in the greater omentum |
| ***Ulcerative Colitis*** | | |
| Oesophageal varices haemorrhage | 47/M | Sudden death due to haemorrhage for oesophageal varices |
| Acute respiratory failure | 54/M | Hypoxaemic during thyroid surgery with acute respiratory distress syndrome, prevented extubation; postoperatively experienced anterior wall myocardial infarction |
| Cardiac arrest | 71/M | Cytomegalovirus colitis, erosive esophagitis, multiple UC exacerbations, and failure to thrive; after prolonged hospitalization expired at nursing facility |

**Supplemental Figure 1. Ustekinumab Exposure Through Up to 5 Years**

**
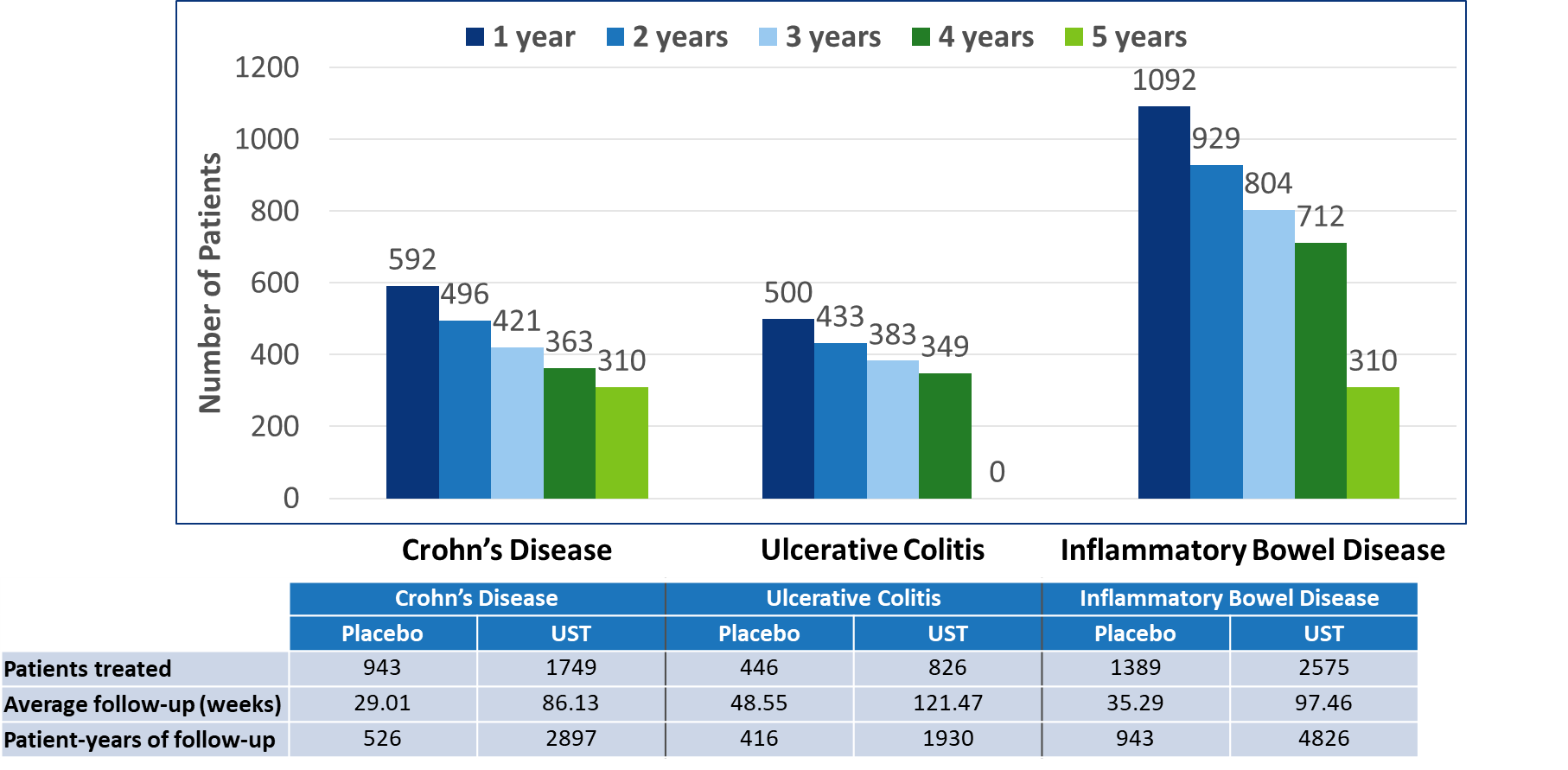
**

**Supplemental Figure 2. Event Rates per 100 Patient-Years for Frequent Adverse Events (A) and Serious Adverse Event (B) Preferred Terms in Inflammatory Bowel Disease (IBD) Group**

**B.**

**A.**

**
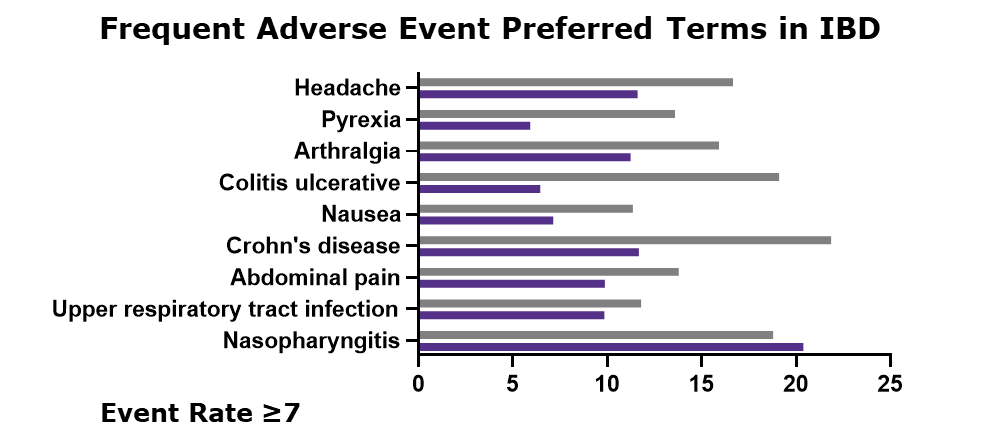

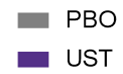
**

**
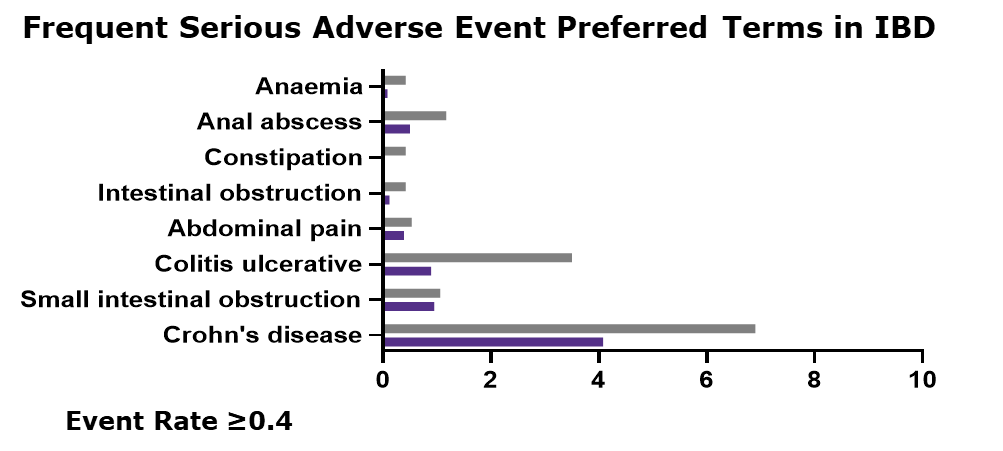
**

PBO, placebo; UST, ustekinumab

**Supplemental Figure 3. Event Rates per 100 Patient-Years for Frequent Infection (A) and Serious Infection (B) by Preferred Terms in Inflammatory Bowel Disease (IBD) Group**

**A.**

**
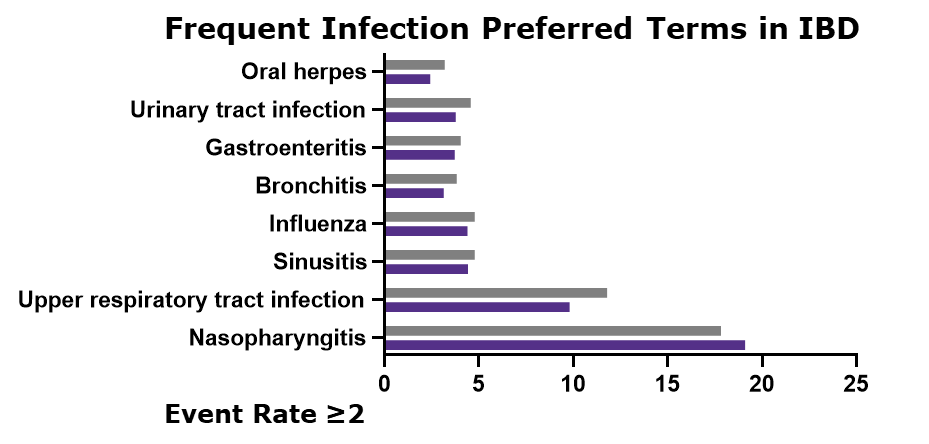

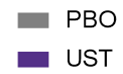
**

**B.**


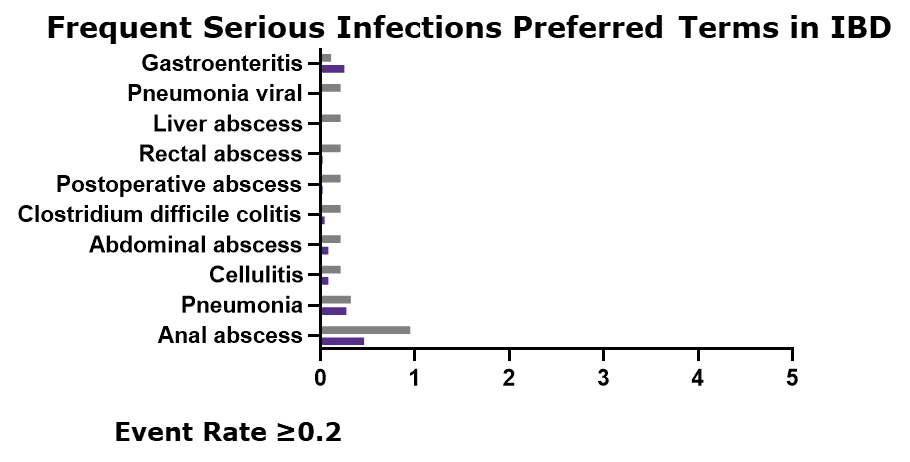


PBO, placebo; UST, ustekinumab
